# LINKS BETWEEN NEIGHBORHOOD CONTEXT AND ACCESS TO SOCIAL CAPITAL IN LATER LIFE

**DOI:** 10.1093/geroni/igz038.245

**Published:** 2019-11-08

**Authors:** Noah J Webster, Kristine J Ajrouch, Toni C Antonucci

**Affiliations:** 1 University of Michigan, Ann Arbor, Michigan, United States; 2 Eastern Michigan University, Ypsilanti, Michigan, United States

## Abstract

Social capital is essential for healthy living in later life, and evidence indicates the importance of neighborhood context in facilitating access to this resource. However, additional research is needed to compare how objective and subjective indicators of context shape this association. We examine links between objective (census tract-level population density and composition) and subjective (perceived safety and quiet) indicators of neighborhoods and social capital (contact frequency, network size, trust others in neighborhood). Data include participants age 65+ (N=259) from Wave 3 (2015) of the longitudinal Social Relations Study based in Detroit, Michigan. Multivariate analyses indicate respondents living in more densely populated neighborhoods reported more frequent contact with network members. Those perceiving their neighborhood as safe reported larger networks, more trust in and greater support exchanged among neighbors. Findings suggest both objective and subjective indicators of neighborhood context shape access to social capital, but perceptions play a larger role.

